# Sleep disturbance, psychiatric issues, and employment status of Iranian people living with HIV

**DOI:** 10.1186/s13104-021-05755-w

**Published:** 2021-08-30

**Authors:** Arezu Najafi, Marzieh Mahboobi, Khosro Sadeghniiat Haghighi, Faezeh Aghajani, Amin Nakhostin-Ansari, Saber Soltani, Ali Jafarpour, Parvin Afsar Kazerooni, Matin Bazargani, Somayeh Ghodrati, Samaneh Akbarpour

**Affiliations:** 1 Occupational Sleep Research Center, Baharloo Hospital, Tehran University of Medical Sciences, Tehran, Islamic Republic of Iran; 2 Department of Epidemiology and Biostatistics, School of Public Health, Isfahan University of Medical Sciences, Isfahan, Iran; 3 Research Development Center, Arash Women’s Hospital, Tehran University of Medical Sciences, Tehran, Iran; 4 Sports Medicine Research Center, Neuroscience Institute, Tehran University of Medical Sciences, Tehran, Iran; 5 Department of Virology, School of Public Health, Tehran University of Medical Sciences, Tehran, Iran; 6 Research Center for Clinical Virology, Tehran University of Medical Sciences, Tehran, Iran; 7 Enter for Communicable Disease Control, Ministry of Health and Medical Education, Tehran, Iran; 8 HIV Expert of Deputy of Health, Tehran University of Medical Sciences, Tehran, Iran

**Keywords:** Sleep quality, Psychiatric issues, Employment, HIV

## Abstract

**Objectives:**

There are limited studies on the psychological issues and sleep problems among the Iranian people living with HIV (HIV). In this study, we aimed to assess sleep disorders, psychiatric characteristics, and employment status among Iranian PLWH.

**Results:**

In total, 304 PLWH with a mean age of 40.01 (SD = 9.60) years participated in the study. About 72% of the participants had a global PSQI score of more than 5, with a mean score of 7.71 (SD = 3.31). About 55.6%, 50%, and 67.4% of subjects had abnormal scores for depression, anxiety, and stress. Unemployed participants had 2.13 times more chance (95% CI 1.01–4.53) of having poor sleep quality compared to employed patients, and stress increased its likelihood by 3.18 times (95% CI 1.47–5.88).

## Introduction

HIV/AIDS has been a major health problem in recent decades, with a heavy burden on the countries’ health systems [1]. Advances in the treatment and management of HIV infection and its aftermath have resulted in increased survival rates for people living with HIV (PLWH), thereby enhancing their quality of life [2]. Over half of HIV-positive people suffer from depression, mania, psychosis, anxiety, and commit suicide. As the disease progresses, physical symptoms such as diarrhea, pain, cough, fever, night sweats, and shortness of breath affect sleep quality. Depression and anxiety are worsened by sleep disturbances, as are the overall quality of life and health [2–4]. Sleep deprivation can also lead to attention deficit disorder, mood swings, fatigue, and cardiovascular complications. In addition, insomnia can reduce treatment response rates, increase disease recurrence and lead to depression [5].

There is evidence that psychological issues are associated with sleep disorders in the general population, but few studies have specifically examined this issue in PLWH. It may be helpful to screen PLWH, as part of routine care, to prevent the progression and side effects of sleep disorders. Therefore, the present study aimed to assess sleep disorders and their associated psychological and socio-economics factors among PLWH.

## Main text

### Materials and methods

A cross-sectional study was conducted between October and November 2019 on 304 PLWH referred to voluntary counseling and testing (VCT) centers in Tehran. A list of the VCT centers from each area of Tehran was compiled, and two were picked at random from each area. In each center, patients were randomly selected and invited to participate in the study. The inclusion criteria were HIV diagnosis, age 18 and older, and giving consent to participate in the study. The study protocol was reviewed and approved by the ethics committee of Tehran University of Medical Sciences (IR.TUMS.VCR.REC.1398.312).

In this study, we used the Pittsburgh Sleep Quality Index (PSQI) and Depression Anxiety Stress Scales (DASS). Six psychologists conducted face-to-face interviews with participants to fill out the questionnaires. The Persian version of PSQI measures sleep quality over the past month, and various studies have confirmed its validity and reliability [6–10]. A total of seven subscales are included in the questionnaire, including (1) sleep duration, (2) sleep disturbance, (3) sleep latency, (4) daytime dysfunction due to sleepiness, (5) sleep efficiency, (6) overall sleep quality, and (7) sleep medication use. Each question can be answered on a Likert scale from 0 to 3, and the total score is calculated from 0 to 21. A total score greater than five indicates poor sleep quality [11].

DASS has three self-reporting scales to assess negative emotional states, including depression, anxiety, and stress. We used the validated Persian translation of the abbreviated version of DASS, consisting of 21 questions, on a Likert scale from 0 to 3 with higher scores indicating more severe depression, anxiety, and stress [12–14].

We also collected patients’ demographic characteristics and medical history, including gender, age, educational level, marital status, history of heart disease, lung diseases, or diabetes, body mass index (BMI), employment status, CD4 count, and co-infection with hepatitis B, C, and tuberculosis. Participants were divided into three groups based on their BMI (less than 25, 25 to 29, and 30 or higher). Employment status was categorized as employed and unemployed. Patients’ last measured CD4 was also recorded.

#### Statistical analysis

There were 14 participants whose PSQI and DASS questionnaires were incomplete and were excluded from the sample. Other missing values (3.11%) were imputed using a single imputation method in R software. We calculated mean and standard deviation (SD) for quantitative variables and frequency and percentage for qualitative variables. A Chi-square test was used to compare qualitative variables, and a T-test was used to compare continuous variables between groups. The logistic Regression model test was used to determine the factors affecting sleep quality. Associations were examined by using the odds ratio and 95% confidence interval. We calculated crude odds ratios for variables, and entered all variables in the model to calculate the adjusted odds ratios. All analyzes were performed with STATA software, and a P-value of less than 0.05 was considered statistically significant.

### Results

In total, 304 PLWH, including 209 men (68.8%) and 95 women (31.2%) with a mean age of 40.01 years, participated in the study. Based on the results of PSQI, 72% of patients had poor sleep quality. Table 1 shows the association between the sleep quality of participants and their demographic characteristics. Although 70% of people with poor sleep quality were men, this difference was not statistically significant. A comparison of other demographic characteristics between the two groups of patients with poor sleep quality and good sleep quality showed that the only significant difference was in their job status, as about 85% of the participants with good sleep quality were employed.

**Table 1:** Sleep quality and its associations with other factors in Iranian PLWH

Variables	Total (n = 304)	Poor sleep quality (n = 219)	Good sleep quality (n = 85)	P-value
	Mean (SD)	Mean (SD)	Mean (SD)	
Age, years (mean ± SD)	40.01 (9.60)	39.80 (9.56)	40.55 (9.22)	0.537
BMI (kg/m^2^)	43.75 (24.73)	24.55 (4.15)	25.19 (3.90)	0.216

	No (Percent)	No (Percent)	No (Percent)	
Sex				
Male	209 (68.8)	151 (68.9)	58 (68.2)	0.904
Female	95 (31.3)	68 (31.1)	27 (31.8)	
Marital status				
Single^a^	149 (49)	106 (48.4)	43 (50.6)	0.732
Married (committed)	155 (51)	113 (51.6)	42 (49.4)	
Education				
Under diploma	146 (48)	41 (48.2)	105 (47.9)	0.975
Diploma	102 (33.6)	29 (34.1)	73 (33.3)	
Upper diploma	56 (18.6)	15 (17.6)	41 (18.7)	
Employment status				
Employed	218 (71.7)	146 (66.7)	72 (84.7)	0.002
Unemployed	86 (28.3)	73 (33.3)	13 (15.3)	

HIV characteristics	Mean (SD)	Mean (SD)	Mean (SD)	
Duration of HIV diagnosed, month (median, IQR)	50 (63.75)	62 (62.5)	49 (64)	0.073
Mean CD4 count (SD)	577.12 (301.21)	580.60 (318.35)	568.15 (253.26)	0.747
Duration of ART, years (Med, IQR)	40.00 (46.75)	47 (47.5)	38.00 (44)	0.241

	No (Percent)	No (Percent)	No (Percent)	
CD4 count				
< 500	135 (44.4)	102 (46.6)	33 (38.8)	0.222
≥ 500	169 (55.6)	117 (53.4)	52 (61.2)	
Route of transmission				
Sexual contact	119 (39.1)	86 (39.3)	33 (38.8)	0.611
Injection drug use	174 (57.2)	127 (58)	47 (55.3)	
Blood products	4 (1.3)	2 (0.9)	2 (2.4)	
Unknown	7 (2.3)	4 (1.8)	3 (3.5)	
HIV/HBV co-infection	9 (3)	7 (3.2)	2 (2.4)	0.697
HIV/HCV co-infection	69 (22.7)	51 (23.3)	18 (21.2)	0.693
HIV/TB co-infection	28 (9.2)	23 (10.5)	5 (5.9)	0.211

^a^Separated and divorced/widowed are in this category

Table 2 shows the association between sleep quality and psychological issues, including stress, anxiety, and depression. The mean PSQI index in participants was 7.71. Among participants, 44% did not have depression at all, while 56% suffered from varying degrees of depression, of which 18% had severe depression. Half of the participants did not suffer from anxiety, and about 20% suffered from very severe anxiety. Also, about 72% of participants with good sleep quality did not have anxiety. About 33% of participants did not have stress, and 11.2% had very severe stress. About 31% of participants with poor sleep quality suffered from mild levels of stress.

**Table 2:** Psychiatric and sleep characteristic of Iranian PLWH

Variables	Total (n = 304)	Poor quality sleep (n = 219)	Good quality sleep (n = 85)	P-value
	Mean (SD)	Mean (SD)	Mean (SD)	
	No (percent)	No (percent)	No (percent)	
Depression				
Normal (0–9)	135 (44.4)	81 (37)	54 (63.5)	< 0.0001
Mild (10–12)	25 (8.2)	17 (7.8)	8 (9.4)	
Moderate (13–20)	63 (20.7)	53 (24.2)	10 (11.8)	
Sever (21–27)	27 (8.9)	22 (10)	5 (5.9)	
Extremely severe (28–42)	54 (17.8)	46 (21)	8 (9.4)	
Anxiety				
Normal (0–6)	152 (50)	91 (41.6)	61 (71.8)	< 0.0001
Mild (7–9)	19 (6.3)	14 (6.4)	5 (5.9)	
Moderate (10–14)	40 (13.2)	34 (15.5)	6 (7.1)	
Sever (15–19)	31 (10.2)	27 (12.3)	4 (4.7)	
Extremely severe (20–42)	62 (20.4)	53 (24.2)	9 (10.6)	
Stress				
Normal (0–10)	99 (32.6)	49 (22.4)	50 (58.8)	< 0.0001
Mild (11–18)	89 (29.3)	68 (31.1)	21 (24.7)	
Moderate (19–26)	45 (14.8)	40 (18.3)	5 (5.9)	
Sever (27–34)	37 (12.2)	33 (15.1)	4 (4.7)	
Extremely severe (35–42)	34 (11.2)	29 (13.2)	5 (5.9)	

Table 3 shows the factors affecting sleep quality in our participants, with only stress and employment status affected the quality of sleep independently. Unemployed patients had a 2.13 times more chance (95% CI 1.01–4.53) of having poor sleep quality than employed patients. Patients’ stress scale score was also associated with their sleep quality (OR = 3.18, 95% CI 1.47–5.88).

**Table 3:** Results of the logistic regression model on the factors affecting the sleep quality in Iranian PLWH

Variables	Crude OR	CI 95% (P-value)	Multivariate adjusted OR	CI 95% (P-value)
Age, years (mean ± SD)	0.99 (0.96–1.02)	0.536	0.99 (0.96–1.02)	0.773
BMI	0.96 (0.90–1.02)	0.216	1.01 (0.93–1.07)	0.973
Sex (female is reference)	1.03 (0.60–1.77)	0.904	1.34 (0.73–2.44)	0.786
Marital status (single is reference)	1.09 (0.66–1.80)	0.732	1.34 (0.73–2.44)	0.335
Education				
Under diploma (reference)	1		1	
Diploma	0.98 (0.561–1.73)	0.952	1.12 (0.57–2.17)	0.736
Upper diploma	1.06 (0.53–2.13)	0.854	1.52 (0.62–3.68)	0.354
Employment status (Employed is reference)	2.76 (1.44–5.32)	**0.002**	2.13 (1.01–4.53)	**0.048**
Duration of HIV diagnosed, month	0.99 (0.99–1.00)	0.121	1.00 (0.99–1.01)	0.747
Duration of ART, years	0.99 (0.98–1.003)	0.241	0.995 (0.98–1.01)	0.441
CD4 count ≥ 500 (CD4 count < 500 is reference)	0.73 (0.43–1.21)	0.223	0.868 (0.46–1.61)	0.868
Route of transmission				
Sexual contact (reference)	1		1	
Injection drug use	1.25 (0.65–2.27)	0.197	1.19 (0.50–2.17)	0.295
Unknown or blood products	1.17 (0.62–1.60)	0.225	1.72 (0.40–3.21)	0.935
HIV/HBV/TB coinfection^a^ (Lack of coinfection is reference)	1.15 (0.65–2.03)	0.615	0.970 (0.46–2.01)	0.970
Depression^b^ (Lack of depression is reference)	2.96 (1.76–4.99)	**< 0.0001**	1.26 (0.61–2.67)	0.537
Anxiety^b^ (Lack of anxiety is reference)	3.57 (2.07–6.15)	**< 0.0001**	1.52 (0.68–3.43)	0.314
Stress^b^ (Lack of stress is reference)	4.95 (2.89–8.47)	**< 0.0001**	3.18 (1.47–5.88)	**0.003**

Bold values indicate variables, which were statistically significant

^a^HIV/HBV or HIV/HCV or HIV/TB co-infection

^b^Mild, moderate, severe, extremely severe were defined as abnormal

### Discussion

There have been many studies demonstrating the effects of drug and alcohol use on sleep quality and sleep disorders, but few have examined the effects of psychological issues on sleep quality, especially in PLWH. Therefore, the present study has examined sleep quality and related factors among Iranian PLWH.

Based on PSQI results, 72% of our participants had poor sleep quality. In studies conducted in Nigeria, Brazil, and Tehran, poor sleep quality was reported in 60%, 47%, and 47.5% of HIV patients, respectively [4, 15, 16]. These variations in the reported prevalence of sleep disturbance among PLWH across studies can be explained by study locations, participants’ demographic characteristics, inclusion and exclusion criteria, or different tools used to collect data.

We did not find any relationship between participants’ gender, age, educational status, marital status, and BMI and their sleep quality, which contrasts with some other studies [4, 17, 18]. Some studies have shown an association between increased BMI and sleep disorders. In people with high BMI, abdominal obesity and difficulty achieving a comfortable sleeping position may contribute to poor sleep quality; however, the reason for this association remains unclear [19, 20]. According to a Chinese study, sleep quality differed according to age, with younger individuals having more sleep disorders due to psychological issues [21].

Almost all components related to sleep quality were significantly different in people with good, and poor sleep quality, similar to previous studies as PLWH typically have sleep disturbances such as reduced sleep hours, waking up at night, and waking up early, overturning short brain waves, and rapid eye movements [22].

Stress negatively impacts sleep quality, as shown in the results. Psychological disorders such as stress and anxiety are leading causes of insomnia among PLWH, and being stressed is more likely to result in difficulty sleeping [23]. Studies on the effects of stress on PLWH show associations between stress and anxiety, depression, sleep quality, and fatigue during the day [24, 25]. Many reasons contribute to HIV-related stress in patients, including fear of infecting others, revealing HIV status, job changes, interpersonal relationships, and changes in personal lives. Long-term stress can reduce their immunity and accelerate the progression of the disease. Providing social support to patients is a proven method of reducing stress and improving health, consequently improving quality of life [26].

In our study, employment status was an independent factor that affected sleep quality, and unemployed people were more likely to have poor sleep quality. Unemployment in PLWH and health-related characteristics were also associated with demographic and social factors, including younger age, lower levels of education, and the presence of stigma in the community [27, 28]. According to Jabbari et al., sleep quality among PLWH was influenced by occupational status and educational level [29]. Poverty is also a chronic stressor that can cause irreparable damage to a person’s physical and mental health, affecting the quality of life, and consequently, sleep quality [30, 31].

### Conclusion

Poor sleep quality seems to be a common issue caused by a variety of factors among PLWH. This group of people needs intervention to improve their sleep quality. Sleep disorders can also be treated with social support, such as finding a job, and non-pharmacological techniques such as sleep hygiene training, behavioral changes, and stress reduction activities.

## Limitations

There are limitations to the present study that need to be addressed in future studies. The first limitation is that the participants were patients from VCT centers in Tehran and may not represent the entire population of Iranians with HIV. Furthermore, as we conducted a cross-sectional study, we could not evaluate the causal relationships between variables. In addition, measuring the quality of life of patients could provide more insight into the effect of sleep quality on people’s lives, which was not investigated in this study.

## Data Availability

The data that support the findings of this study are available from the corresponding author upon reasonable request.

## Abbreviations

PLWH: People living with HIV
PSQI: Pittsburgh Sleep Quality Index
DASS: Depression Anxiety Stress Scales
BMI: Body mass index
